# A Bacillus of Scurvy

**Published:** 1899-02

**Authors:** 


### A Bacillus of Scurvy.

Prof. Babes describes a bacillus which produces the gingivitis and hemorrhages of scurvy. He observed sixteen Roumanian soldiers who, after exhaustion due to hard work, living under the same conditions of locality and diet, all feel ill at nearly the same time, a favorable point toward the infectious nature of the disease. The clinical symptoms varied but slightly in the different cases; the gums were notably tumefied, and around the teeth formed a swollen, fungous, hard, reddish blue or livid white ridge. The superficial strata of these ridges were ulcerated and covered with a pseudo-membranous layer of a dirty yellow color. The slightest pressure caused bleeding.

Prof. Babes found a characteristic bacillus, elongated, curved, pointed at the ends, 0.3$\mu$ in width and 3$\mu$ in length; occasionally it presents itself as a much longer rod, or in the form of undulating filaments of various sizes. The younger specimens have the form of diplo-bacteria, and from the beginning of their development show a tendency to produce chromatic corpuscles, which take on an intense stain with methylene-blue, and which show themselves at the ends of the bacilli, or are distributed at equal intervals over their whole length. Upon agar it develops in extensive colonies of a yellowish hue, mixed with other colonies, chiefly streptococci. It is interesting to note that a pure culture could only be obtained by transplanting material taken from these yellow colonies, after a brief vegetation in common with the streptococci, in glycerin-agar in which the streptococcus had been cultivated for some time. It is hence probable that this coccus prepares a favorable soil for the existence of the bacillus. This result was constant in all the cases, and inoculation constantly caused copious hemorrhages in rabbits. The author believes that this bacillus is one of those usually found in the human mouth, for he has often observed similar ones in the peridental deposits. It is probable that in weakened individuals it may assume a pathogenic power which it does not normally possess.—*Archives de Medecine Experimentale, No.* 9.

